# Cytotoxicity Evaluation of Proroot MTA, Root MTA and Portland Cement on Human Gingival Fibroblasts

**Published:** 2007-10-02

**Authors:** Mohammad Reza Sharifian, Mehrnoosh Ghobadi, Noushin Shokouhinejad, Hadi Assadian

**Affiliations:** 1 *Department of Endodontics, School of Dentistry, Dental Research Center, Tehran University of Medical Sciences, Tehran, Iran*; 2 *Endodontist, Private practice, Tehran, Iran*; 3 *Department of Endodontics, School of Dentistry, Tehran University of Medical Sciences, Tehran, Iran*

**Keywords:** Cytotoxicity, Mineral Trioxide Aggregate, Portland Cement

## Abstract

**INTRODUCTION:** The aim of this study was to compare the cytotoxicity of ProRoot MTA (PMTA), Root MTA (RMTA) and Portland cement (PC) on human gingival fibroblasts (HGFs).

**MATERIALS AND METHODS:** The extracts of the test materials were placed close to human gingival fibroblasts (HGFs) within 96-well plates. Cellular viability was assessed using MTT assay in different intervals (freshly mixed, 4, 24, and 168 hours after mixing). The data were analyzed using the One-way ANOVA and Tukey’s test at 95% significance level.

**RESULTS:** It was indicated that there was not a significant difference in cytotoxicity of test materials (p>0.05). In addition, there was not a statistically significant difference between different time intervals within each group (p>0.05).

**CONCLUSION:** PMTA, RMTA and PC showed comparative biocompatibility while evaluated *in-vitro*.

## INTRODUCTION

Mineral trioxide aggregate (MTA) was introduced in 1993 in Loma Linda University as a root-end filling material (1-2). MTA is a biocompatible material with numerous clinical applications in endodontics. Clinical applica-tions include: pulp capping, internal/external root-resorption repair procedures, perforation repairs, and root end fillings (3-4).

MTA (ProRoot MTA) is marketed in gray-colored (GMTA) and white-colored (WMTA) preparations, both containing 75% Portland cement, 20% bismuth oxide, and 5% gypsum by weight (5). It has been shown that after periradicular surgery with MTA, periapical lesions heal to an almost normal condition (6). Biocompatibility is the ability of a material to perform an appropriate host response in a specific application. This means that in contact with materials, the tissue of the patient does not suffer from any toxic, irritating, inflammatory, allergic, genotoxic, or carcinogenic action (7-8).

Herein, the biocompatibility of MTA has been investigated in many studies through bioassays both in-vivo and in-vitro (5, 9-14).

Recently, there has been a great interest in the evaluation of Portland cement as an alternative to MTA, because Portland cement costs less and is widely available. Several studies have compared MTA with Portland cement (PC) and the findings suggest that they seem almost identical macroscopically, microscopically, and by X-ray diffraction analysis (15-16). It has been shown that there is no significant difference between the dominant compounds in both WMTA and White PC except the presence of bismuth oxide in WMTA (15).

Other studies affirm that Portland cements contain the same chemical elements and similar properties as MTA (17-19). The biocompati-bility of Portland cement has been previously investigated in-vitro using a number of cells, including mouse lymphoma cells (20), human endothelial cells (21), Chinese hamster ovary cells (22), and human osteosarcoma cells (23). This suggests that Portland cement has the potential to be used as a less expensive root-end-filling material in dental practice (24). However, these cell lines have an aneuploid chromosome pattern, and the cells multiply rapidly with an unlimited life span. Therefore, the results obtained from these cells might differ from those obtained in real human tissue.

Recently, a material similar to ProRoot MTA (PMTA) was developed by Lotfi in Iran named Root MTA (RMTA) which was claimed to have the exact properties of PMTA. Different studies investigated the properties of RMTA in comparison with PMTA. In various in vivo and in vitro investigations, it has been proven that RMTA has so many similarities with PMTA (25-31).

But to date, there has been no comparison of PMTA, RMTA and PC regarding cytotoxicity of these materials on Human Gingival Fibroblasts (HGFs) that involve in the healing and/or regeneration of periradicular tissues. Therefore, the purpose of this study was to investigate the cytotoxicity of PMTA, RMTA and PC on HGFs using MTT assay.

## MATERIALS AND METHODS

In this in vitro study, human gingival fibroblasts (HGFs) were taken from Pasteur Institution Cell Bank (Pasteur Institution, Tehran, Iran). Cells were grown in RPMI 1640 cell culture containing 10% bovine fetal serum, antibiotic and antifungal agents. Then the cells were cultured in 96-well plates and incubated for 24 hours (5% CO2, 37ºC). After sterilizing in dry heat at 160ºC for 2 h, gray ProRoot MTA (Dentsply, Tulsa, OK, USA) , Root MTA (Salamifar, Tehran, Iran) and Portland cement (Simane Tehran Co., Tehran, Iran) were mixed in a powder/distilled water weight ratio of 3/1 in sterile Petri dishes and placed in artificial root models.

In order to simulate the application of the test materials and their setting conditions with in vivo status, modified Socorex tips (Socorex Isba S.A., Switzerland) were used. Therein, after end-resection of these non toxic plastic cones in a certain place, plastic cones of 4mm in diameter at the tips were obtained. Then, the items were sterilized by autoclave. After placing the plastic cones on a sterile glass block from the cut-end, 5 millimeter-thick materials were placed inside them and compacted partially by a plugger and a sterile moist cotton pellet was placed on the materials.

Then the total complex was placed in 96-well plates containing 20 µL of culture media. In order to prepare the extracts of the mixing time, the complex was taken out of the well, some minutes after the placement and the culture of the well was taken for granted as the extract of the mixing time.

In order to prepare the extracts of 4, 24 and 168 h following mixing, the extracts were transferred to cultured cells at the bottom of the plate. Because this study was not designed for observing a dose response relationship, the material extracts were not diluted. After 24 h of extracts incubation, cellular viability was evaluated using MTT assay. A culture medium devoid of any examination material extract was used as control.

In order to perform MTT assay, a stock MTT solution (5 mg/mL) was prepared as follows: Fifty mg MTT powder was added to 10 mL PBS. To prepare the final MTT solution (0.5 mg/mL), 1ml stock solution was added to 9 mL PPMI containing 5% FCS and antibiotic. After 24 h of close contact between the extracts and cells, the culture media containing the extracts were emptied on cells, 200 mL of the final MTT solution was added to each group and the plates were incubated for 3 h. After this period, MTT solution was removed from the cells and 200 µL isopropanol was added to each group to dissolve formazan crystals. After performing liquid pipettage for each group, optic density (OD) in 450 nm wavelength was read by ELISA reader (STAT FAX. 2100, USA) and recorded thereafter. Optical absorption is positively related to the number of metabolically active cells. The data were analyzed using the One-way ANOVA and Tukey’s test at 95% significance level.

## RESULTS

Optic density values of MTT assay is shown in Table 1.

The results of cytotoxicity evaluation of the examined materials in different time intervals showed no significant difference among the test materials, and between them compared with the negative control group (p>0.05).

In addition, there was not a significant difference between different time intervals in cytotoxicity of each group (P>0.05).

## DISCUSSION

In present study, the cytotoxicity of PMTA, RMTA and PC on HGFs was investigated. The 3-(4, 5-dimethylthiazol-2-yl)-2, 5- diphenyl tetrazolium bromide (MTT) assay was used in this study. The MTT assay focused on the capacity of mitochondrial dehydrogenizing enzymes in living cells to convert the yellow water-soluble tetrazolium salt into dark blue formazan crystals. The water insoluble product is stored in the cytoplasm of living test cells. The amount of formazan formed is directly related to the mitochondrial enzyme activity (32).

In this study, cellular assessment of test materials was done by material extracting method (32-33). The extracts were prepared in four different times. The purpose of preparing the extract at the mixing time was preparing probable toxic products from the freshly mixed material. The aim of preparing extracts 4 hours after mixing was that within this period the material was being set and most of the chemical reactions were supposed to happen within this period of time. Preparing the extracts 24 and 168 h after mixing was aimed at cytotoxicity evaluation after the setting process. In this study, the direct method was not utilized but the material extracts were used. The reason was the limitation in placing the artificial models containing the materials on the cells for 168 h. In a pilot study, the cells present in 96-well plates which were in direct contact with the materials within the plastic cones could only remain viable for 3-4 days and none of the cells within any of the groups survived at any time there after. This could have affected the results of the study. Taking this into consideration and also in order to evaluate the effect of these materials on the surrounding as well as distant cells (33), the material extracts were utilized. After application of this method in the pilot study, the total death following direct contact with test materials was not observed.

**Table 1:** The mean and standard deviation of optic density (OD) for each group at 4 different periods

	**Freshly mixed**	**4 hours**	**24 hours**	**168 hours**
**PMTA**	0.285± 0.021	0.328± 0.044	0.319± 0.079	0.276± 0.059
**RMTA**	0.328± 0.112	0.343± 0.131	0.286± 0.076	0.266± 0.057
**PC**	0.314± 0.064	0.341± 0.158	0.320± 0.102	0.255± 0.049
**control**	0.289± 0.039	0.376± 0.041	0.292± 0.062	0.243± 0.031

In this study, the maximal OD in all groups was seen 4 hours after mixing. Thereafter, OD value In this study, the maximal OD in all groups was seen 4 hours after mixing. Thereafter, OD value decreased 24 hours after mixing. The minimal OD value was seen, 168 hours after mixing. In another study evaluating cytotoxicity of PMTA and RMTA on L929 fibroblast cell line (30), the average cellular viability in 168 h was less than that of 48 and 72 h, corresponding with the results of the current study. The reason for decreasing cellular viability in 24 and 168 h compared with that of 4 h is unknown. Likewise, in this study, there was no significant difference in cellular responses between MTA and PC. RMTA also showed favorable results.

## CONCLUSION

PMTA, RMTA and PC showed comparative cytotoxicity on human gingival fibroblasts.
